# Supplementary material for: S-Nitrosylation of Tissue Transglutaminase in Modulating Glycolysis, Oxidative Stress, and Inflammatory Responses in Normal and Indoxyl-Sulfate-Induced Endothelial Cells
Source: Int J Mol Sci. 2023 Jun 30;24(13):10935. doi: 10.3390/ijms241310935 (PMC10341700; doi:10.3390/ijms241310935)
Supplement: Supplementary file 1 [file ijms-24-10935-s001.zip › ijms-2458200-supplementary.pdf]

Supplemental Table and Figures

Supplemental Table S1: Antibodies used in this study.

Antibody Name	Company	M.W. (kDa)
ACE	Santa Crus (sc-23908)	195
Fibronectin	Abcam (ab6328)	220
HO1	Abcam (ab68477)	33
PKM2	Cell Signaling (#4053)	60
GAPDH	Sigma-Aldrich (G8795)	37
TGM2 cub7402	Labvision, Thermo Fisher	80
G6PD	ThermoFisher (MA5-15918)	59
CCN1	Santa Crus (sc-374129)	40
IκBα	Cell Signaling (#9242)	39
NFκB	Invitrogen (PA5-27617)	65
eNOS	Abcam (ab76198)	133
Phospho-eNOS	Cell Signaling (#9571)	140
β-actin	Novus bioloogicals (NB600-501)	42
2° Ab		
Goat Anti-Mouse	Jackson Immuno (115-035-003)	
Goat Anti-Rabbit	Jackson Immuno (111-036-045)	

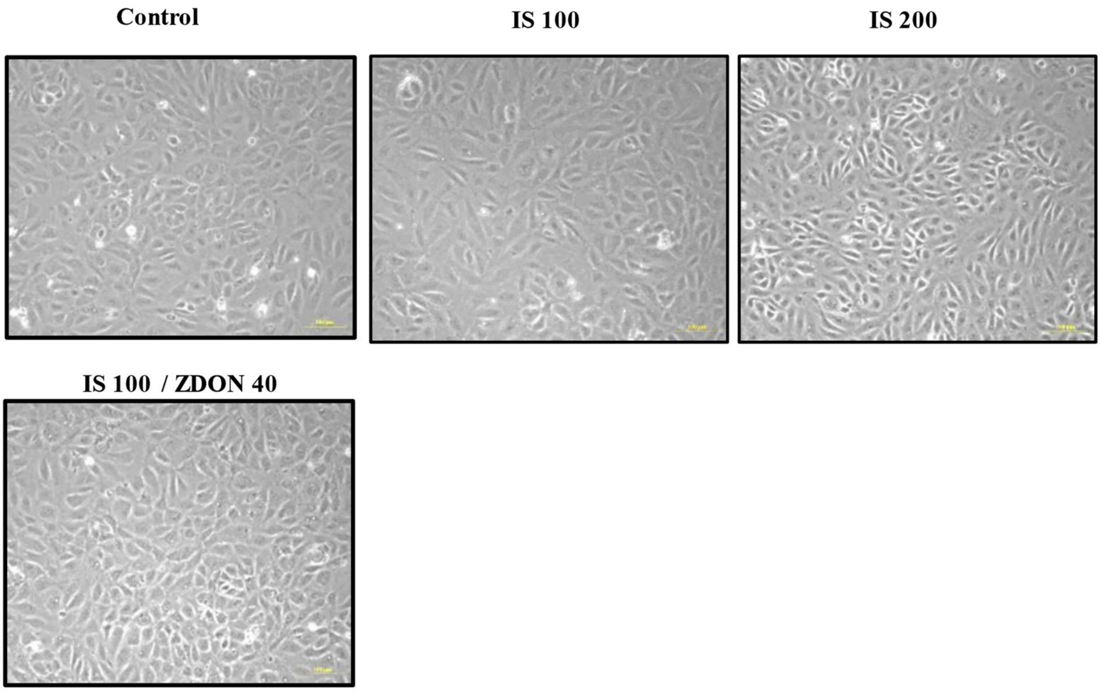

Supplemental Figure S1. Morphology of cells after treated with chemicals or/and IS. HUVEC cells (passage 2-3) were grown to confluent on the 6-well dish. The morphology of HUVEC cells pretreated with either ZDON (40 μM) followed by 4 hours of incubation with IS (100, or 200 μM and imaged under light microscopy (200 x).

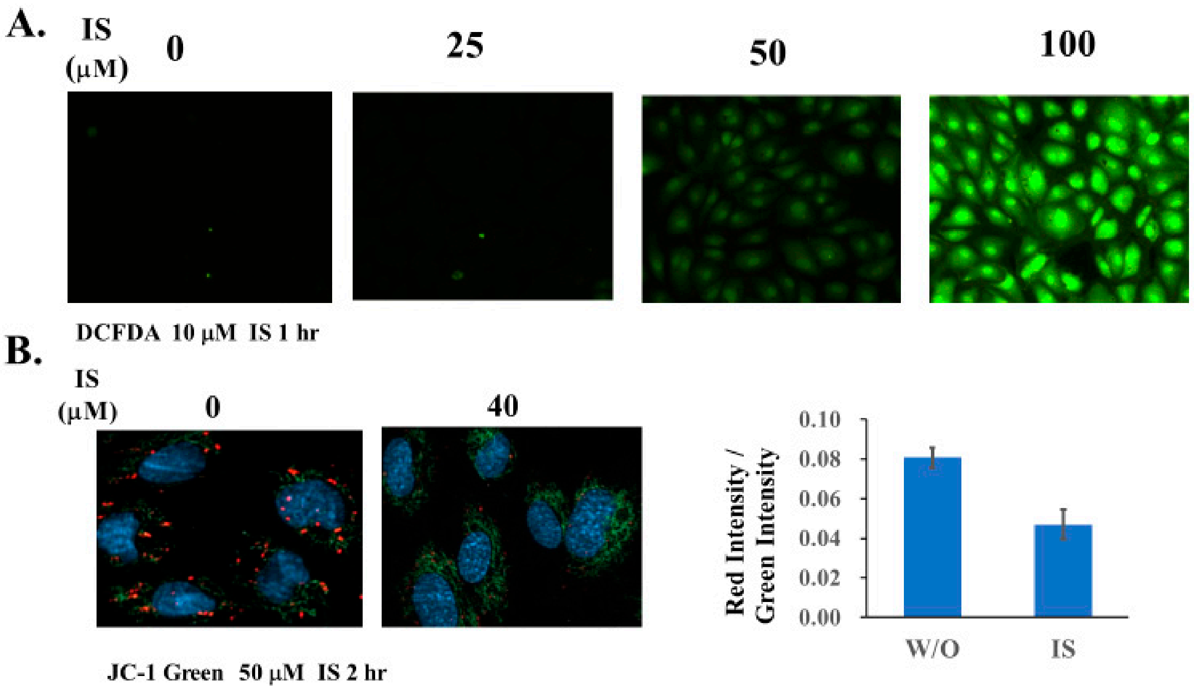

Supplemental Figure S2. Reactive oxygen species (ROS) generation and mitochondrial dysfunction upon IS exposure. HUVEC cells were pre-incubated with of 10 μM DCFH-DA before being treated with IS (0, 25, 50 and 100 μM) for 1 hr. **(A)**. The ROS generation was examined using fluorescence microscope visualized with FITC filter. **(B)** The mitochondria's function were examined using Molecular Device's Image Pico plate reader. Cells were pretreated with 1 μM JC-1 Green and 0.1 μM Hoesch 33342 before being treated with 50 μM of IS for 2 hr. The intensities of green (represent drysfunctional mitochondria) and red (represent functional mitochondria) fluorescence were quantified and normalized for number of cells.
